# Influences of specialty identity when implementing a new emergency department in Denmark: a qualitative study

**DOI:** 10.1186/s12913-024-10604-0

**Published:** 2024-02-01

**Authors:** Jeanette Wassar Kirk, Mette Bendtz Lindstroem, Nina Thórný Stefánsdóttir, Ove Andersen, Byron J. Powell, Per Nilsen, Tine Tjørnhøj-Thomsen

**Affiliations:** 1 Department of Clinical Research, Copenhagen University Hospital Hvidovre, 2650 Hvidovre, Denmark; 2 Department of Health and Social Context, National Institute of Public Health, University of Southern Denmark, Odense, Denmark; 3 Emergency Department, Copenhagen University Hospital Hvidovre, 2650 Hvidovre, Denmark; 4 Faculty of Health and Medical Sciences, Department of Clinical Medicine, University of Copenhagen, 2200 Copenhagen, Denmark; 5 Center for Mental Health Services Research, Brown School and School of Medicine, Washington University in St. Louis, St. Louis, MO USA; 6 Department of Health, Medicine and Caring Sciences, Linköping University, Linköping, Sweden; 7 School of Health and Welfare, Halmstad University, Halmstad, Sweden

**Keywords:** Specialty identity, Oilcloth sessions, Qualitative study, Interviews, Ethnographic field study, Implementation science

## Abstract

**Background:**

The Danish Health Authority recommended the implementation of new types of emergency departments. Organizational changes in the hospital sector challenged the role, identity, and autonomy of medical specialists. They tend to identify with their specialty, which can challenge successful implementation of change. However, investigations on specialty identity are rare in implementation science, and how the co-existence of different specialty identities influences the implementation of new emergency departments needs to be explored for the development of tailored implementation strategies. The aim of this study was to examine how medical specialty identity influences collaboration between physicians when implementing a new emergency department in Denmark.

**Methods:**

Qualitative methods in the form of participants’ observations at 13 oilcloth sessions (a micro-simulation method) were conducted followed up by 53 individual semi-structured interviews with participants from the oilcloth sessions. Out of the 53 interviews, 26 were conducted with specialists. Data from their interviews are included in this study. Data were analysed deductively inspired by Social Identity Theory.

**Results:**

The analysis yielded three overarching themes: [1] ongoing creation and re-creation of specialty identity through boundary drawing; [2] social categorization and power relations; and [3] the patient as a boundary object.

**Conclusions:**

Specialty identity is an important determinant of collaboration among physicians when implementing a new emergency department. Specialty identity involves social categorization, which entails ongoing creation and re-creation of boundary drawing and exercising of power among the physicians. In some situations, the patient became a positive boundary object, increasing the possibility for a successful collaboration and supporting successful implementation, but direct expressions of boundaries and mistrust were evident. Both were manifested through a dominating power expressed through social categorization in the form of in- and out-groups and in an “us and them” discourse, which created distance and separation among physicians from different specialties. This distancing and separation became a barrier to the implementation of the new emergency department.

## Background

In many western countries, organizational changes in the hospital sector have challenged the role, identity and autonomy of medical professionals [1]. In 2007, policy reforms by the Danish Health Authority recommended the implementation of new types of emergency departments (EDs) with one common entrance for all acute patients (in Danish “Fælles Akutmodtagelser”) [2]. One of the most ground-breaking recommendations for the new EDs was the continuous presence of senior physicians at the frontline. Previously, nurses and trainee physicians conducted the triage of patients, and specialty physicians were called in based on the type of injury or disease. The idea of placing senior physicians at the frontline was based on the assumption that collaboration between medical specialties would improve diagnosis and treatment [3]. When the policy reform was presented, the emergency medicine (EM) specialty was not established; it was established a decade later [4]. Previously physicians from other medical specialties were responsible for handling the admission, treatment, and discharge of acute care patients. The establishment and implementation of an EM specialty have resulted in the training of physicians and nurses in emergency medicine for employment at the ED. The intention is to enhance the skills, coordination, and response to emergencies among healthcare professionals in the ED, thereby improving patient outcomes in critical situations.

Medical specialists tend to identify with their specialty, emphasizing key features of their specialty, such as certain expertise, values, norms, beliefs, attitudes, and behaviours [5]. Medical specialties have specific professional identities and domains of knowledge that can strengthen the diagnosis and treatment of patients within the specialty. Being a member of a specialty can improve self-esteem and provide social organization, support and understanding in challenging times [6]. However, these characteristics can also hinder collaboration between specialized departments within the hospital [6]. Research has shown that inter-physician conflicts can have a negative impact on physicians’ well-being and professional performance [7–11]. According to Social Identity Theory (SIT) [12], people derive part of their self-concept and sense of self-esteem from group memberships. Their self-view is not determined by personality characteristics or by their inter-personal relationships alone. People’s self-view is also determined by the groups to which they belong, that is, their social identity [5]. However, divisions in social identities can have a negative effect on relationships if and when specialties become siloed. A potential side-effect is that one specialty implicitly puts another specialty down [6] and they imagine the worst in each other, which can exacerbate inter-group conflict [13]. Further, research has shown that silos in medicine also adversely affect patients due to poor communication and lack of information-sharing across disciplines, which can lead to medical errors [6] and stifle the adoption and use of safer practices [8]. Therefore, the collaboration of various specialty identities along patient pathways is of utmost importance for the well-being of both the patient and healthcare professionals. This social identity approach ensures that different medical practices are not viewed as isolated functions separated by time and space, as emphasized by Huzzard et al. [9].

In a previous study, we found that EM physicians experienced a lack of recognition, acknowledgement and support from other medical specialties. In some cases, they felt they were met with mistrust and criticism of their expertise, e.g. doubt that they can handle specialty patients when they are admitted acutely [10]. Other studies have documented similar findings, e.g. Pedersen et al. [11] observed how EM physicians in a Danish ED experienced mistrust from various medical specialties.

Tailored implementation strategies may be required to establish and implement new EDs effectively and address the influences of different specialty identities [14]. However, specialty identity has rarely been investigated in implementation science [15, 16] and the development of tailored implementation strategies [14] needs to explore how the co-existence of different specialty identities influences the implementation of new EDs. To complement the knowledge gap in implementation science regarding specialty identity as a determinant (factors of importance for the implementation, e.g. barriers and facilitators) in implementation processes and support the implementation of new EDs, the aim of this study was to examine how medical specialty identity influences collaboration between EM physicians and physicians from other medical specialties when implementing a new ED in Denmark.

## Methods

### Analytical perspectives

The analytical point of departure of this study is based on empirical data and the results from two previous studies evaluating an implementation strategy called oilcloth sessions (a micro-simulation method) used in Denmark as part of the implementation of new EDs [17, 18]. As previously mentioned, the data revealed group conflicts among different medical specialties during their participation in these oilcloth sessions. This inspired us to explore specialty identity deductively through an analytical lens inspired by SIT [5]. Inspired by Freidson [12], specialty identity refers to the unique and professional identity that develops within a specific field, such as medicine. Specialty identity is shaped by the norms, values, and the specific knowledge base that characterize the profession. According to Freidson, professionals often seek to maintain a degree of autonomy and control to preserve their professionalism and protect their professional domain [12]. The representatives in this study were physicians from the EM specialty and physicians from other medical specialties (e.g. cardiology, gastroenterology and gynaecology).

### Social identity theory

SIT focuses on the nexus between the individual and the group. It explores how people see themselves and others in terms of social categories and how that affects our perceptions, attitudes, and behaviour [5]. According to SIT, social identity within a group can lead to group members dividing social groups into an in-group (us) and an out-group (them) and group members of an in-group will seek to find negative aspects of an out-group, thus enhancing their self-image [5]. SIT highlights that inter-group conflicts and competition can be triggered solely by the awareness of different groups, and prestige is the result of social comparison [5]. An integral part of social identity is trust and distrust among members of the group and towards members of other groups. Power is immanent in all social relations, according to Foucault et al. [19], who state that power is manifested through discourses, which take the form of a specific type of knowledge or language. This enables certain things to be articulated while restricting or disallowing the expression of others. Applying SIT and the concept of power in our analysis allows us to focus on the ways different medical specialties create social categorizations and manifest power and explore the consequential impacts on the collaboration and implementation of the new ED.

An ethnographic field study was carried out to examine how specialty identity influenced collaborations among EM physicians and physicians from different medical specialties when implementing a new ED in Denmark. Ethnographic field studies are well suited to study the meaning that participants attribute to phenomena in their naturalistic settings and the social interactions between participants [20, 21]. The context for this examination is the observation of 13 oilcloth sessions [22, 23] used to support the implementation of a new ED [17].

In addition, we conducted follow-up semi-structured interviews with 53 participants from the oilcloth sessions. Out of the 53 interviews, 26 were conducted with specialists. Data from the interiwievs with the specialists were included in this study. This enabled us to explore how participants experienced the oilcloth sessions and elaborate on the situations that we observed. Semi-structured interviews were chosen because they enabled us to subtly push forward the conversation, so prepared questions were covered while also allowing interesting topics to arise during the interviews [24].

### Oilcloth sessions

Oilcloth sessions are an educational implementation strategy [25] with the purpose of developing new patient pathways amd training staff, key employees and managers in these new patient pathways and introducing a new physical building design. A key employee is defined as an employee appointed by managers to play a central role in the implementation of a new ED in their department [17]. Staff, managers and key employees work together on a blueprint with plastic figures representing ED staff on a scale of 1:50 to generate knowledge and workplace learning about the planned implementation. The sessions were carried out in a 30-m^2^ room in the hospital. All participants and the facilitator stood around a table to view the blueprint. Each oilcloth session lasted between 1.5 and 2 hours. Kirk et al. [17] have described the oilcloth method in detail. Oilcloth sessions allow for discussions of, for example, new forms of organization, physical layout in relation to which specialties should be placed where and which spaces should be allocated to whom, and methods of communication.

### Study setting

The study was carried out at a large urban university hospital in Denmark with 700 beds, 5000 employees, and around 100,000 admissions a year within a catchment area of 550,000 citizens. Establishing the new ED will involve different types of organizational change, e.g. several specialist departments providing bed capacity and physicians to the new ED on two separate floors, moving to a new building with new facilities and layout, and the continuous presence of medical specialists in the ED. Further, the number of beds in the ED bed section will increase from 26 to 92 beds with stays of up to 48 h, and a short-stay unit with stays of up to 6 h will be introduced. Approximately 200 employees work in the current ED, which is expected to increase to 275 in the new ED. The new ED will open in spring 2024.

### Participants

On average, 15 to 20 participants participated in each oilcloth session. The participants consisted of the hospital’s board of directors, managers and key employees from different professions and positions, and they represented different acute and medical specialist departments (Table 1). In total, 64 hospital staff participated in the oilcloth sessions.

**Table 1:** Participating departments

Specialty	Department
Medical specialty	Cardiology
	Gastroenterology (Medical)
	Infectious Diseases
	Internal Medicine (including Respiratory Medicine and Endocrinology)
Surgical specialty	Orthopaedic Surgery
	Gastroenterology (Surgical)
Emergency specialty	Emergency Department
Other	Clinical Biochemistry
	Obstetrics and Gynaecology
	Paediatrics and Adolescence Medicine
	Radiology

### Data collection

Data included field notes from the ethnographic study of the 13 oilcloth sessions carried out by NTS and JWK between May 2019 and October 2020. Observations were used to understand and interpret collaboration episodes and situations among EM physicians and physicians from other medical specialties when implementing the new ED. At each observation, an observation matrix divided into three columns was used: [1] observations; [2] reflections; and [3] analytical remarks. Both oral utterances and body language were considered. At the end of a session, additional notes, reflections, analytical concepts and remarks were added. Reflections and central points from the field notes were discussed continuously with the rest of the author group [17]. The field notes from the observations resulted in 315 single-spaced pages.

After the oilcloth sessions, interviews were conducted between October 2019 and December 2020 by JWK and NTS. They were held in the offices of the participants or meeting rooms at the hospital. On average, the interviews lasted 40 min (from a minimum of 26 min to a maximum of 53 min) and covered eight themes contained in a semi-structured interview guide (See Stefánsdóttir et al. [10] for published interview guide). The interview guide was developed based on field notes and reflections from the observational study. Thus, the interviews aimed to verify our findings from the observational study and address considerations about specialty identity that emerged along the way. The interview guide was developed by NTS and JWK and was pilot-tested with a senior consultant employed at the management secretariat of the hospital. This was conducted by JWK, and NTS wrote notes and asked follow-up questions. The pilot test led to minor revisions of the interview guide. All interviews were recorded and transcribed verbatim by a research assistant, resulting in 403 single-spaced pages.

For this study, the data consisted of semi-structured interviews conducted with the 26 physicians who participated in the oilcloth sessions. They were employed in 12 different departments. The participants included 10 chief physicians, 13 senior physicians and three trainee physicians. To ensure anonymity, participants are presented as physicians from or representatives of their specialty within four categories: emergency, medical, surgical, and other specialty, and not according to their position.

### Data coding and analysis

The field notes from the observations were read and re-read by MBL to get a sense of the whole. The a priori codes “us and them”, “knowledge elite” and “power” were chosen based on social identity theory to guide deductive coding processes. The code “us and them” reflects the boundaries between one’s own specialty (“us”) and other specialties (“them”) intended to protect the specialty’s knowledge and authority and to retain control and autonomy over the specialty’s practices. The code “knowledge elite” applied to situations when representatives from one specialty emerged as dominant by asserting themselves scientifically or as professional experts and thereby acted or functioned as a “knowledge elite” vis-à-vis other specialties. The code of “power” was manifested through discourses, which took the form of a specific type of knowledge or language.

An initial deductive coding of the transcribed field notes guided by SIT was conducted by MBL [26]. Then, a secondary in-depth deductive analysis of both the field notes and the interview data was conducted by JWK. Data were condensed in a coding scheme divided into meaning units and then abstracted on a manifest level meaning coding close to the text. Data condensation refers to the process of reducing and simplifying the collected data to identify patterns and themes in the gathered material [27]. Quotes were chosen to ensure a clear link between the data from field notes and the interviews to strengthen the confirmability of the findings [24]. JWK, OA, MBL and NTS read and discussed the coding scheme until agreement was reached to strengthen the validity of the analysis. Then, JWK abstracted the coded material on a latent level [28].

The main findings were discussed in meetings with the rest of the author group (Table 2). These meetings were conducted as structured sessions where JWK facilitated the discussion regarding the analytical results. An example of how these meetings contributed to refinements of analytic outputs was the selection of a series of quotes that were particularly pronounced in relation to the code ‘us and them.’ Collectively, we decided to exclude these highly impactful statements from the manuscript for ethical reasons, as we did not want to be co-agents in reinforcing and reproducing too extreme examples of an ‘us and them’ dynamic between different specialties.

**Table 2:** Sub-themes and themes

Sub-themes	Themes
Boundary drawing	Ongoing creation and re-creation of specialty identity through boundary drawing
In-group and out-group	
Tacit knowledge	
Collective re-creation of boundaries	
Us and them as social categorization	Social categorization and power relations
Power struggles	
Us and them as discourses	
Change in boundaries	The patient as a boundary object
The patient as a mediating artefact	

### Ethical considerations

The study was approved by the Danish Data Protection Agency (ID no. VD-2019-160). In accordance with Danish law, formal ethical approval is not mandatory for studies not involving biomedical issues and was therefore not obtained. The study adhered to the ethical principles of the Helsinki Declaration and oral and written informed consent was obtained from all participants. With inspiration from situational ethics, our knowledge of the organizational context of the hospital was handled with responsibility, morality and intuition during the observations and interviews [29]. For example, we made the conditions of anonymity clear and underlined our independence from the hospital management as a part of the introduction to each oilcloth session and interview. Optimal anonymity was ascertained by assigning participants codes instead of using their names in the field notes and transcriptions.

## Results

The analysis yielded three overarching themes: [1] ongoing creation and re-creation of specialty identity through boundary drawing; [2] social categorization and power relations; and [3] the patient as a boundary object.

### Theme 1: Ongoing creation and re-creation of specialty identity through boundary drawing

The analysis showed that specialty identity was constituted by social dynamics and alliances between physicians from the same specialty, which in turn created boundaries against physicians from other specialties. Boundaries are social constructs that delineate which knowledge or meaning systems are deemed relevant in interactions [30]. These boundaries became apparent in prevailing narratives during the oilcloth sessions and interviews. The narratives from physicians from the medical specialties occasionally provided evidence that they perceived their knowledge as superior to that of the EM physicians. An example of this was during a discussion about the investigation of patients with stomach pain:

*“As specialists, we possess specialized knowledge to handle the patients. Specialized knowledge that we do not believe physicians from the emergency department have to the same extent.” [interviews, physician no. 11]*.

Many of the specialty boundaries were made explicit in episodes and discussions dealing with diagnostics, referral of patients to the specialist departments and handling of specific patient cases in the ED. Here, specialized procedures performed by EM physicians were questioned by other specialist physicians. For example, an EM physician explained:

*“Well, it wasn’t that long ago that some of our colleagues from a specialist department criticized our professional skills. They didn’t think we were skilled enough.” [interviews, physician no. 17]*.

The analysis showed that a specialty was made explicit through boundary drawing in terms of what was considered expertise, which thus legitimated participation in the specialty. Specialty boundaries were also formed by using internal concepts and professional abbreviations. A discussion between a group of surgical physicians and EM physicians proceeded as follows:

*“Again, it is discussed, which physician should attend to the patient and when. A physician from the surgical department thinks the patient should be attended to and diagnosed within four hours and prepared for surgery. A physician from the EM specialty asks why this patient should not be evaluated within 30 minutes by a specialist, [which] is the instruction by the Danish Health Authority and decided by the board of directors. The physicians from the surgical department reject this and do not believe it is relevant to this patient as it is ‘an obvious patient’. ” [field notes, oilcloth session no. 6]*.

This is one of many cases where specialist physicians used internal concepts and professional abbreviations. An “obvious patient” was understood as a patient who exhibits classic and thus recognizable symptoms, diagnoses or diseases classified within the specific specialty. This, in turn, constituted specialty boundaries in relation to other specialties. Thus. a statement such as “an obvious patient” referred to a high degree of tacit knowledge within the surgical specialty. The use of tacit knowledge becomes a way of creating social categorizations in the specialty (the in-group) while distinguishing themself from the other specialties (the out-group). Drawing these specialty boundaries includes groups, activities and language and a way to maintain stability in the specialty identity and exclude others.

### Re-creation of boundaries

Boundaries were not stable but were re-created. When discussions focused on challenges with external partners outside the hospital (e.g. paramedics or general practitioners), the physicians and managers, irrespective of the medical specialty, collectively moved closer together and thereby re-created their specialty boundaries. One example from the field notes was:

*“The departmental managers do not trust the quality of pre-hospital visitation. It becomes even more important that we work together.” [field notes, oilcloth session no. 2]*.

Collective re-creation of boundary drawing against external partners constituted a sense of unity among EM physicians and physicians from the medical specialties. Thereby, a new in-group was created as the physicians collectively discussed negative aspects of the external partners (an out-group). That situation is an example of specialty identity as an unstable formation where the group of physicians regardless of specialty expanded their group identity and boundaries were dissolved. This was particularly evident if both the EM physicians and physicians from the specialist departments experienced the benefits and prestige of this alliance. A physician from the medical specialty stated:

*“It would be fantastic if the general practitioner became better at specifying the admission diagnosis. It would be helpful for you in the emergency department, but also for us as medical physicians, as we would then have a common starting point” [field notes, oilcloth session no. 3]*.

### Theme 2: Social categorization and power relations

The data revealed several examples of specialist physicians expressing boundaries against EM physicians. For example, discussions arose about which specialty-related procedure or physical locations belonged to whom, highlighting differences among specialties emphasizing what is “ours” (the in-group) and demonstrating power towards another specialty (the out-group). In several oilcloth sessions, discussions arose about who could claim which rooms and why. A specialist physicians expressed:

*“We need two rooms for preliminary examinations. It is a central procedure in our specialty. It is not the kind of procedure that characterizes the EM specialty. So, the rooms must belong to us.” [field notes, oilcloth session no. 3]*.

The analysis showed that social categorization was voiced when the responsibilities of the specialties were,up for discussion during the oilcloth sessions, e.g. when the representatives of the EM physicians made proposals for changing a clinical procedure. The physicians from the specialist departments either refused to accept their suggestion with the message *“it’s our patient”* or *“this patient’s treatment is our responsibility”* or at best did not comment on the suggestions made. Such a situation is described in the following extract from the field notes:

*“[it is discussed] which patients should be placed in the surgical section of the ED, a manager from a surgical [specialty] considers making a ‘positive list’ for the flow coordinator. A member of the board of directors points out that the flow coordinator controls the allocation of physicians to the patients. This statement from the board of directors’ triggers tumult and resistance […] and a discussion regarding the flow coordinator’s option of relocating the medical resources [follows]. In the surgical department, there is no consensus on whether it should be like this– they want to see ‘their patients’.” [field notes, oilcloth session no. 2].*

The positive list mentioned in the quotes above can be viewed as a mediating artefact to support *“who owns the patient”*, as well as to maintain a specialty’s power and control over management and specialty rights. Other examples were disagreements about setting quality objectives and how to achieve them. In many examples, there were divergent views on the treatment and care of specialty patients. These negotiations arose as oilcloth sessions became a space where different specialty identities, socio-cultural norms as well as assumptions that could be taken for granted and power relations embedded in these identities unfolded.

As part of the implementation of the new ED, the flow coordinator was to be an EM physician, who would be in charge of triaging patients to the right specialties and allocating physicians. At an oilcloth session, this was met with frustration among the specialty physicians. One surgical specialty physician questioned whether it was even possible to discuss the flow coordinator function:

*“Can we [even] discuss the flow coordinator function? [He answers the question himself:] No, it’s not like that. Okay, who made that decision? Because if it is a decision made by the board of directors [it is] fine, but… can[‘t] we ask each other if it is rational or what? We can easily allocate our own resources and maintain the overview of our patients.” [field notes, oilcloth session no. 3]*.

These types of pronounced frustrations occurred several times during the oilcloth sessions when the argumentation against the flow coordinator was linked together with ways of securing “high quality” in the patient pathways. This quality referred to achieving patient safety, securing flow and following standards. However,,in a perspective of SIT the frustrations could also be understood as efforts to protect one’s specialty identity, demonstrating or trying to hold on to power and maintain prestige. The physicians doubted that the flow coordinator could assess whether patients should be seen by a specialist and maintain an overview of the new ED and the specialists’ whereabouts and activities. The specialist physicians were reluctant to hand over the management of the specialized patients and their time and priority to the flow coordinator. Thereby, the top-down decisions with organization of the new ED by a flow coordinator challenged existing workflows and autonomy for the physicians from the specialist departments, which became visible through social categorization, power demonstration and specialty boundaries in the form of statements as “*we can*”, “*own resources*” and “*our patients*”.

In some situations, physicians from the EM specialty succeeded in convincing physicians from the specialist departments of certain ways of handling the patient pathways or organizing the ED. An example was given by an EM physician, who reported several negotiations about the mandate to lead the implementation process of the new ED and thereby the mandate to make the final decisions:

*“There has been a lot of resistance from several of the specialists regarding organizational issues. We have tried to seek consensus and slowly achieve consensus on the agreements we make. It seems like we have started to succeed in convincing some of the specialists, so they have begun to show more trust in us as managers and our organizational decisions, e.g. some of the physicians from the surgical specialty have begun to understand that it is not to bother them that they cannot undertake surgery when they are on duty in the ED, because their expertise is important for the patients who are admitted with, for example, abdominal pain.” [interviews, physician no. 17]*.

The example relates to discussions about organizational issues pertaining to the implementation of the new ED that were repeatedly problematized. These issues related to uncertainty about the distribution of the specialist department’s *“own”* procedures, medical resources, the allocation of *“own”* and *“others”* patients in *“their”* section and freedom to plan their own tasks and procedures without interference from the flow coordinator, with the risk of diluting the power of the specialty. All situations where physicians and managers from different specialties exposed social categorization were a sign of power struggles and sometimes distrust between the different specialties. This complicated the collaboration and the implementation of the new ED.

The analysis revealed that power is embedded in the different specialties’ identities, which was expressed through actions where specialist physicians ensured that decisions were made in their interests or with a background in knowledge representing their specialty; e.g. explicitly (“we have been assigned the responsibility”) or (“we possess specialized knowledge to handle the patients”) or more subtly (by not commenting on suggestions) or by expressing distrust in the overall objectives. Thus, power was not something that one specialty owned or had but was expressed through social relationships in the tension field between physicians from different specialties and their specialty identities.

### Theme 3: The patient as a boundary object

Although boundary drawing and power were evident in the analysis, which predominantly conveyed the impression of stable specialty identities and networks of relations, “the patient” as an object (including patient pathways) sometimes changed these boundaries and formations. The patients and patient pathways were discussed in great detail by the physicians from the specialties and the EM physicians. They discussed who could and should handle treatment responsibilities for *“their patient”*. The discourses of “*our or their patient*” related to expert knowledge, which provided certain ways of acting that partly only physicians within the specialty understood but also encompassed a more general knowledge and an opportunity to strengthen an early collaboration:

*“I think we all know the situations where we are called [to the ED] to take care of a patient admitted with a heart attack [all physicians nod]. Many of us [specialist physicians] have experienced that it strengthens the patient pathways if we are specialist physicians from different specialties from the beginning (with the possibility of quickly clarifying differential diagnoses).” [field notes, oilcloth sessions no. 3]*.

Another example was when the physicians discussed the best and most relevant treatment for the patient. An EM physician argued:

*“We all want to treat the patient with […] high quality, that is the case. We sometimes have different perspectives about what is the right treatment. It depends on the specialty. But in these oilcloth sessions, we try to negotiate and learn from each other even if we have to cross clinical boundaries.” [interviews, physician no. 22]*.

In these examples, the patient served as a collaborative bridge connecting various specialties, knowledge and their respective identities. In this manner, the patient was transformed into a boundary object [30], which could be comprehended by physicians from multiple specialties, an object that was flexible enough to accommodate the unique identities and local requirements of each specialty, while also being resilient enough to uphold a shared identity across all specialty identities. This became evident in the many discussions between physicians from the medical specialties and the EM physicians about whether the EM physicians should handle treatment responsibilities for *“their patient”.* In these discussions, the patient becomes an effective boundary object establishing a common language and knowledge for physicians with different specialty identity backgrounds [30]. Even though the analysis shows reasonably stable social categorizations between specialties, the patient as a boundary object opens another possibility for a “softening” of these stable social categorizations. This was shown as a means to communicate concerns or questions about patient treatments or pathways across boundaries. Through this, the participants were empowered to transform their own knowledge in the light of the adoption of the new ED, which supported the implementation of the new ED.

In other situations, “the patients” acted as an ineffective boundary object, which was expressed through criticism and distrust in other physicians’ knowledge and competencies. An EM physician explained:

*“They [the specialists] think they are experts in everything, while our knowledge as EM physicians is doubted. But what do they know about patients with co-morbidities, for example?” [interviews, physician no. 9]*.

In the patient cases discussed during the oilcloth sessions, physicians from specialist departments were concerned about who was the right and first physician to review the patients. One discussion was about a clinical case of a young woman with stomach pain. It was discussed which specialty she was to be referred to. A physician from one of the specialties expressed that the patient had to be referred directly to her department and explained:

*“If it turns out that it is not something [relevant to our specialty], we will send the patient back to the ED and then the [other specialty] can take over.”*

An EM physician responded:

No, that will not work. We cannot have the patients drifting between different departments. You need to arrange joint conferences with the [other specialist] physicians, and you will be the one to seek out the patient.

The physician then replied:

*“If we assume that the physician from the surgical department is the first physician to review the patient, it will increase the quality rather than if it is an EM physician and thereby also the ‘hit rate’ of whether it is a patient who needs to be referred to our department.” [field note, oilcloth session no. 7]*.

Other situations concerned the vision for the new ED and dealt specifically with the articulation of the specialist departments’ ability and willingness to innovate and change attitudes. An EM physician expressed:

*“Right now, some of the physicians from the specialist departments have a strong resistance against the overall objectives of the new ED. I feel like they only want to maintain their current ways of working and are not open to seeing opportunities to make our patient pathways even better.” [interviews, physician no. 9]*.

The resistance from the other specialties is interpreted by the EM physician as a way of trying to change or adapt the overall objectives of the new ED to benefit their socio-cultural practices and specialty identity. Being critical was a way of boundary drawing and dividing specialty physicians and EM physicians into “in- and out-groups” and thereby exercising power. Thereby, specialty identities, negatively impacted on the collaboration between different physicians with different specialty identities, which complicated the implementation of the new ED.

The analysis showed that specialty identity has an important influence on collaboration between physicians. This is evident in the explicit delineation of boundaries and implications of social categorization that entails ongoing creation and re-creation of boundary drawing and exercising power between the EM physicians and the other specialty physicians. In some situations, “the patient” as a positive boundary object affected the collaboration in a positive direction, but direct expressions of boundaries and mistrust were evident. Both were manifested in a dominating power expressed through a discourse of an “us and them,” which created distance and separation between specialties.

## Discussion

Using the implementation strategy oilcloth session, we examined the influence of specialty identity on collaboration and implications for the implementation of a new ED. Our main findings showed that specialty identity became an important collective determinant in relation to the implementation of the new ED.

First, specialty identity was established as both implicit and explicit boundary drawing, revealing itself as social categorization expressed through different specialist knowledge, jurisdictions and distrust. A study by Abbott and Meerabeau [31] showed that professions tend to defend their jurisdictions fiercely when experiencing incursions. The same processes unfolded in our study between the physicians from different specialties in the oilcloth sessions. The need to collaborate in new ways and roles triggered reactions where the boundaries of expertise were sharply defined, for example, through the clarification of specific professional knowledge. Boundaries are becoming more explicit because of increasing specialization but being a member of a specialty is not necessarily negative but can improve social organization and self-esteem and provide support in challenging times [6]. The main problem with strong specialty identities is when such divisions become siloed. This happens when networks of relationships constituting a specialty and the knowledge embedded within this close in on themselves. Møllekær et al. [32] showed that specialties close in on themselves to protect their position of power and preserve their self-esteem. This can be problematic for collaboration and members of siloed specialty identities can imagine the worst in members from other specialties, which can lead to inter-group conflicts [13].

In contrast to Kanjee and Bilello [6], our results showed that specialty identity was not only siloed but was also not a stable formation. Specialty identity was an unstable formation depending on the context and developed in an ongoing creation and re-creation process through social categorization of “us and them” (in- and out-groups) depending on whether the physicians experienced an enhanced self-image and prestige of this alliance. Levine and Reicher [33] demonstrated that this fluidity in social identity can provide hope for changing the collective behaviour and avoiding intractable identity conflicts [13] between physicians from the EM specialty and from other specialties.

Intractable identity conflicts are defined as intense, deadlocked, and resistant to resolution. They have two characteristics. First, they have a win-lose element [13]. This element was evident in the oilcloth sessions during discussions about physical locations. Further, it seemed that the EM physicians struggled for a position in the medical specialty hierarchy. This was visible in strong boundary drawing by physicians from other specialties demonstrated by statements that distrusted the EM physicians’ competencies and organizational powers, e.g. the flow coordinator function. A process described by Molleman and Rink [34] focuses on how members of new specialties feel pressure to legitimize their own unique expertise to members of other specialties and especially members of specialties with a strong social identity [35]. They often experience identity threat when confronted with members of new specialties because they may have to reposition their own domain because of this development. The second characteristic is the high stakes. Participants are likely to settle if the stakes are low [36]. Our results showed that the stakes were perceived high by all specialties, which became visible through the power struggles that unfolded during the oilcloth sessions. This is a characteristic of intractable identity conflict where participants use the powers available to them to prevail and maintain specialty prestige.

Second, power played an important role in collaboration among physicians and in maintaining specialty identity, and it was not something that physicians from one specialty “owned” or had. Rather it was expressed through the discourses of “us and them” and in social relations displaying distrust, particularly towards external collaborators and towards the knowledge and competencies of EM physicians. As mentioned by Foucault et al. [19], power is immanent in all social relations and is expressed through discourses in the form of a particular kind of knowledge or language that allows some things to be said and disallows others. This type of power manifestation (e.g. using professional abbreviations) was prominent at the oilcloth sessions and further triggered the possibility for intractable identity conflicts among different physicians from different specialties. The literature suggests that training focused on inter-personal skills, such as giving and receiving feedback, can increase collaboration among professions with strong specialty identities [34]. This type of intervention could be relevant when applying oilcloth sessions as an implementation strategy, because developing new patient pathways implies different specialist physicians receiving feedback from colleagues from other specialties. This resulted in several of the physicians experiencing identity threat when others asked them to justify their actions.

Tackling collaboration problems requires a clear and assertive manager, who can set demands, express expectations and delineate what is acceptable and unacceptable. Mayer et al. [37] highlight the management’s role in shaping the values and norms among employees, and managers should be able to influence relationships between physicians who struggle with different specialty identities. It would require managers who are skilled in group dynamics and conflict management [35]. Our results showed strong resistance from physicians from other specialties against the overall objectives of the new ED, demanding assertive management, built on positive psychology [38]. According to Barlebo Rasmussen [39], the challenge is that many managers of professional organizations, including the healthcare sector, struggle with a laissez-faire leadership style, which results in unclear communication about expectations to the employees. This can lead to collaboration problems between engaged experts with strong specialty identities turning into conflicts. In our study, this also led to obstacles to the successful implementation of the new ED. One tool for handling sharp boundary drawing and conflicts in the collaboration among physicians from different specialties is constructive confrontation, which focuses on moving away from the unrealistic goal of resolution and instead focuses on how these conflicts can be conducted more constructively [36]. Constructive confrontation refers to a communication strategy in which individuals address conflicts, disagreements, or challenging situations in a positive and solution-oriented manner.

Third, we found that even though boundary drawing, power struggles and mistrust were visible in the oilcloth sessions among the participants, the patient became a boundary object [30] by bridging collaborations among the physicians regardless of specialty and thereby changed the boundaries and power formations. The patient as a boundary object has been described before in the literature. For example, Keshet et al. [40] described how the symptoms of the patients became boundary objects between staff from complementary medicine and surgery. Some symptoms were present pre-operatively, whereas others occurred post-operatively. Therefore, the knowledge and competencies of both specialties were necessary to ensure the quality of the patients’ pathways and treatment. The same patterns were visible in the oilcloth sessions, where the opportunity for physicians from different specialties to contribute their professional knowledge to the patient’s treatment was sometimes well received among all the participants. This collaboration took new patient pathways in a positive direction, by developing new patient pathways which was one of the goals of the sessions.

The results of the study appear to be transferable to other contexts and professions beyond the healthcare sector. Within educational research, Edwards [41] has focused on the importance of building common knowledge at the boundaries between practices among professions, such as psychologists and social workers, if the quality of child/youth interventions is to be ensured. Our results could contribute to in-depth knowledge of how specialty identity among different professionals enhances or complicates such collaborations.

At times, specialty identity complicates the collaboration among physicians in our study, especially when the discourse is marked by criticism of and distrust in others’ knowledge and competencies. Thereby, “the patient” became a non-effective boundary object. These situations were related to the discourses on claiming ownership over the patients by talking about “our patients”. The idea of some specialties “owning” special conditions or diseases and patients becoming an artefact symbolized part of their specialty identity.

The essential significance of the concept of “ownership”, also defined as “responsibility-based medicine” [42], is evident in medical education; the progressive development of a sense of ownership over patients under the physician’s care has always been a crucial measure in evaluating trainees’ readiness for eventual independent practice [43]. At its essence, patient “ownership” entails a physician’s dedication to approaching each patient with a profound sense of personal responsibility, ensuring that the patient’s healthcare outcomes are optimized according to their specific circumstances. This commitment involves fully accepting and embracing their role in providing care for the patient [11]. Thus, ownership as part of specialty identity can be desirable to ensure high-quality patient care, especially when talking about patients with a single disease or injury. The challenge is that globally, there has been an increase in patients who have more than one diseases [43] and the prevalence of multimorbidity is increasing [44]. Thus, the boundaries between the medical specialties become more and more permeable and collaboration becomes more necessary.

Permeable specialties imply that it rarely makes sense to talk about a single “owner” but multiple “owners” to ensure high quality throughout the patient’s pathway and treatment both in the new ED and in the healthcare system in general. It requires physicians from different specialties to collaborate, including EM physicians and physicians from other specialties, to avoid potential negative outcomes for the patients in the form of unclear coordination or discussions on the goals of treatment, the patient’s desires and wishes and potential benefits and harms.

### Contribution to implementation science

Our results show that specialty identity has an important collective influence on collaboration among physicians when implementing a new ED. Thus, medical specialty can be seen as an implementation determinant, defined as barriers and facilitators that can hinder or enhance implementation [45]. Implementation science often has a strong focus on individual determinants [45–47] and tends to give less attention to more collective determinants, often referred to as contextual determinants (e.g. organizational culture and climate) [48]. The difference between specialty identity and culture is that specialty identity is constructed externally through membership, whereas culture is based on socially constructed categories that teach us ways of being and includes expectations for social behaviour or ways of acting [49]. Thus, culture becomes internally constructed and much more ingrained and fundamental.

In implementation research, there is an emerging focus on the importance of installing and utilizing trust among stakeholders when implementing organizational changes [50, 51]. This was also an issue in this study where trust and distrust were an integrated part of specialty identity and establishing trust among physicians cultivated a more positive attitude supporting the implementation of a new ED.

The intention with implementation science is identify and develop strategies to address the determinants of implementation [22, 25]. Our results also contribute with the strategy of constructive confrontation [36] as a strategy for handling boundary drawing and conflicts among members with different specialty identities. As part of Powell et al.’s [22, 23, 52] compilation of discrete implementation strategies, constructive confrontations fit in as part of the planning strategies that emphasize the importance of recruiting or securing certain types of managers for change effort, especially because change efforts often elicit resistance.

### Limitations and trustworthiness of the findings

One limitation of the study was our sole focus on the physicians as representatives for the specialty. A focus solely on the physicians emerged from our several years of experience following the implementation of the new ED, where structures for physicians’ work (specialists at the forefront) have generated the most resistance over time [10, 17, 18]. Consequently, physicians were represented most prominently in the oilcloth sessions. By incorporating the perspective of nurses on specialty identity, we might have gained additional insights into the significance of specialty identity for the implementation of a new ED.

A strength of the study was the use of two qualitative methods to collect data: ethnographic fieldwork and semi-structured interviews. This allowed us to compare interview data with data from the field study conducted as part of the oilcloth sessions. This enhances validity because some informants may respond with answers that they believe the researchers want to hear rather than expressing their honest opinions. The comparison enabled us to treat the data as a whole rather than fragmented. NTS and JWK are both experienced qualitative researchers with extensive backgrounds in conducting ethnographic field studies and interviews, which was a strength of the study and helped to reduce bias. The results from our ethnographic fieldwork contributed with knowledge about complex adaptive systems in the form of specialty identity, which is a requirement in implementation science [53].

## Conclusion

The study showed that specialty identity is an important determinant of collaboration among physicians when implementing a new ED. Specialty identity involves social categorization and entails ongoing creation and re-creation of boundary drawing and exercising of power between the EM physicians and the other specialty physicians. In some situations, the patient became a positive boundary object increasing the possibility for a successful collaboration, which supports a successful implementation, but direct expressions of boundaries and mistrust were evident. Both were manifested through a dominating power expressed through a social categorization in the form of in- and out-groups but also sometimes in a discourse of an “us and them”, which created distance and separation between physicians from different specialties. This distancing and separation became a barrier to the implementation of the new ED, because collaboration, willingness to take risks and working towards a common future in collaboration between specialties were limited. This threatened the overall political objective of the implementation of new EDs with improved visitation, diagnostics, treatment and collaboration.

## Data Availability

The data set generated and analyzed during the current study is not publicly available as it contains potentially identifying or sensitive information that could compromise the privacy of the respondents according to regulations set out by the Danish Data Protection Agency but is available from the corresponding author on reasonable request.

## Abbreviations

ED: emergency department
EM: emergency medicine

## References

[1] Salvatore D, Numerato D, Fattore G (2018). Physicians’ professional autonomy and their organizational identification with their hospital. BMC Health Serv Res December.

[2] The Danish Health Authority. Enhanced emergency health service - recommendations for regional planning. 2007. Copenhagen: The Danish Health Authority; 2007.

[3] Sundhedsstyrelsen. Anbefalinger for organisering af den akutte sundhedsindsats - Planlægningsgrundlag for de kommende 10 år. 2020;144.

[4] Sundhedsstyrelsen. Vurdering af et speciale i akut-medicin i Danmark [Internet]. 2017. Tilgængelig hos: 978-87-7104-902-2.

[5] Tajfel H, Turner JC, Austin WG, Worchel S (1979). An integrative theory of intergroup conflict. The Social Psychology of Intergroup Relations Monterey.

[6] Kanjee Z, Bilello L, Leadership. & Professional Development: Specialty Silos in Medicine. J Hosp Med [Internet]. 1. juni 2021 [henvist 23. december 2021];16(6). Tilgængelig hos: https://www.journalofhospitalmedicine.com/jhospmed/article/240388/hospital-medicine/leadership-professional-development-specialty-silos. 10.12788/jhm.3647 34129488

[7] Horwitz LI, Meredith T, Schuur JD, Shah NR, Kulkarni RG, Jenq GY (2009). Dropping the Baton: a qualitative analysis of failures during the transition from Emergency Department to Inpatient Care. Annals of Emergency Medicine Juni.

[8] Paine LA, Baker DR, Rosenstein B, Pronovost PJ (2004). The Johns Hopkins Hospital: identifying and addressing risks and Safety issues. Joint Comm J Qual Saf Oktober.

[9] Huzzard T, Ahlberg BM, Ekman M (2010). Constructing interorganizational collaboration: the action researcher as boundary subject. Action Res September.

[10] Stefánsdóttir NT, Nilsen P, Lindstroem MB, Andersen O, Powell BJ, Tjørnhøj-Thomsen T, editors. m.fl. Implementing a new emergency department: a qualitative study of health professionals’ change responses and perceptions. BMC Health Serv Res. december 2022;22(1):447. 10.1186/s12913-022-07805-w PMC8985264 35382815

[11] Pedersen AHM, Rasmussen K, Grytnes R, Nielsen KJ (2018). Collaboration and patient safety at an emergency department– a qualitative case study. JHOM 19 Marts.

[12] Freidson E (1988). Profession of Medicine: a study of the sociology of Applied Knowledge [Internet]. Afterword W a new, redaktør.

[13] Fiol CM, Pratt MG, O’Connor EJ (2009). Managing intractable identity conflicts. AMR Januar.

[14] Baker R, Camosso-Stefinovic J, Gillies C, Shaw EJ, Cheater F, Flottorp S. m.fl. Tailored interventions to overcome identified barriers to change: effects on professional practice and health care outcomes. I: The Cochrane Collaboration, redaktør. Chichester, UK: John Wiley & Sons, Ltd; 2010. [henvist 16. september 2015]. 10.1002/14651858.CD005470.pub2. Cochrane Database of Systematic Reviews [Internet]. 10.1002/14651858.CD005470.pub2 PMC4164371 20238340

[15] Duncan EM, Francis JJ, Johnston M, Davey P, Maxwell S, McKay GA. m.fl. Learning curves, taking instructions, and patient safety: using a theoretical domains framework in an interview study to investigate prescribing errors among trainee doctors. Implement Sci. 2012;7(1):86. 10.1186/1748-5908-7-86 PMC3546877 22967756

[16] Murphy M, McCloughen A, Curtis K. Using theories of behaviour change to transition multidisciplinary trauma team training from the training environment to clinical practice. Implementation Science [Internet]. december 2019 [henvist 30. april 2020];14(1). Tilgængelig hos: https://implementationscience.biomedcentral.com/articles/10.1186/s13012-019-0890-6. 10.1186/s13012-019-0890-6 PMC6489197 31036023

[17] Kirk JW, Stefánsdóttir NÞ, Powell BJ, Lindstroem MB, Andersen O, Tjørnhøj-Thomsen T (2022). Oilcloth sessions as an implementation strategy: a qualitative study in Denmark. BMC Med Educ 23 juli.

[18] Kirk JW, Stefansdottir NT, Andersen O, Lindstroem M, Powell BJ, Nilsen P. m.fl. How do oilcloth sessions work? A realist evaluation approach to exploring ripple effects in an implementation strategy. 10.1108/JHOM-01-2023-0022 PMC11346207 38825598

[19] Foucault M, Foucault M (2020). Power. Faubion JD, redaktør.

[20] Hammersley M (2006). Ethnography: problems and prospects. Ethnography and Education Marts.

[21] Kirk JW, Haines ER, Ethnography. I: In: Nilsen, P, Birken, SA, editors Handbook on implementation science. Edward Elgar Publishing, Northampton.; 2020. s. 480–7.

[22] Powell BJ, McMillen JC, Proctor EK, Carpenter CR, Griffey RT, Bunger AC (2012). m.fl. A compilation of strategies for implementing clinical innovations in Health and Mental Health. Med Care Res Rev April.

[23] Powell BJ, Waltz TJ, Chinman MJ, Damschroder LJ, Smith JL, Matthieu MM. m.fl. A refined compilation of implementation strategies: results from the Expert recommendations for Implementing Change (ERIC) project. Implementation science [Internet]. December 2015 [henvist 21. juni 2017];10(1). Tilgængelig hos: http://implementationscience.biomedcentral.com/articles/10.1186/s13012-015-0209-1. 10.1186/s13012-015-0209-1 PMC4328074 25889199

[24] Denzin NK, Lincoln YS. redaktører. The SAGE handbook of qualitative research. 5th edition. Los Angeles London New Delhi Singapore Washington DC Melbourne: SAGE; 2018. 968 s.

[25] Proctor EK, Powell BJ, McMillen JC (2013). Implementation strategies: recommendations for specifying and reporting. Implement Sci.

[26] Bingham AJ, Witkowsky P. Deductive and inductive approaches to qualitative data analysis. Analyzing and Interpreting Qualitative data: After the Interview. 2021;133–46.

[27] Mason J. Qualitative researching. 3rd edition. Thousand Oaks, CA: SAGE Publications; 2017.

[28] Lindgren BM, Lundman B, Graineheim UH. Abstraction and interpretation during the qualitative content analysis process. Int J Nurs Stud. 2020;(108):1–6. 10.1016/j.ijnurstu.2020.103632 32505813

[29] Tjørnhøj-Thomsen T, Samværet. Tilblivelse i tid og rum. I: Hastrup K, redaktør. Ind i verden. København: Hans Reitzels Forlag; 2003. s. 93–117.

[30] Star SL (1989). Work(s): JRGR. Institutional Ecology, translations and Boundary objects: amateurs and professionals in Berkeley’s Museum of Vertebrate Zoology, 1907-39. Soc Stud Sci.

[31] Abbott pamela, Meerabeau L. The sociology of the Caring professions. 2.Edition. Routledge; 1998.

[32] Møllekær A, Duvald I, Obel B, Eskildsen J, Kirkegaard H. december. The organization of Danish emergency departments may not have allowed for a full realization of their performance potential. Scand J Trauma Resusc Emerg Med. 2015;23(S1):A52, 1757-7241-23-S1-A52.

[33] Levine M, Reicher S. Making sense of symptoms: self-categorization and the meaning of illness and injury. Br J Social Psy Chology. 1996;(35):245–56. 10.1111/j.2044-8309.1996.tb01095.x 8689097

[34] Molleman E, Rink F (2015). The antecedents and consequences of a strong professional identity among medical specialists. Soc Theory Health Februar.

[35] Molleman E, Broekhuis M, Stoffels R, Jaspers F (2008). How Health Care Complexity leads to Cooperation and affects the autonomy of Health Care professionals. Health Care Anal December.

[36] Burgess H, Burgess G (1996). Constructive confrontation: a transformative approach to intractable conflicts. Mediation Q juni.

[37] Mayer DM, Aquino K, Greenbaum RL, Kuenzi M (2012). Who displays ethical Leadership, and why does it Matter? An examination of antecedents and consequences of ethical Leadership. AMJ Februar.

[38] Seligman MEP. Authentic happiness: using the new positive psychology to realize your potential for lasting fulfillment. Atria paperback edition. New York London Toronto Sydney New Delhi: Atria Paperback; 2013. 321 s.

[39] Barlebo Rasmussen S. Potentialeledelse. 2015.

[40] Keshet Y, Ben-Arye E, Schiff E (2013). The use of boundary objects to enhance interprofessional collaboration: integrating complementary medicine in a hospital setting: integrating complementary medicine in a hospital setting. Sociol Health Illn Juni.

[41] EDWARDS A. Relational agency in professional practice: A CHAT analysis. 2007 [henvist 23. august 2013]; Tilgængelig hos: http://kuir.jm.kansai-u.ac.jp/dspace/handle/10112/7572.

[42] Hansson SO (2022). Responsibility for health. Cambridge, United Kingdom New York, NY, USA Port Melbourne, Australia New Delhi.

[43] Kimberly McLaren K, Lord J, Murray SB, Levy M, Ciechanowski P, Markman J (2013). m.fl. Ownership of patient care: a behavioural definition and stepwise approach to diagnosing problems in trainees. Perspect Med Educ 23 April.

[44] Juul-Larsen HG, Christensen LD, Bandholm T, Andersen O, Kallemose T, Jørgensen LM (2020). m.fl. Patterns of Multimorbidity and differences in Healthcare Utilization and complexity among acutely hospitalized medical patients (≥ 65 Years)– A latent class Approach. CLEP Februar.

[45] Nilsen P, Birken SA (2020). redaktører. Handbook on implementation science.

[46] Estabrooks CA, Floyd JA, Scott-Findlay S, O’Leary KA, Gushta M (2003). Individual determinants of research utilization: a systematic review. J Adv Nurs.

[47] Légaré F, Ratté S, Gravel K, Graham ID (2008). Barriers and facilitators to implementing shared decision-making in clinical practice: update of a systematic review of health professionals’ perceptions. Patient Educ Couns Dec.

[48] Powell BJ, Mettert KD, Dorsey CN, Weiner BJ, Stanick CF, Lengnick-Hall R (2021). , m.fl. Measures of organizational culture, organizational climate, and implementation climate in behavioral health: a systematic review. Implement Res Pract Januar.

[49] Hasse C (2014). An anthropology of learning: on nested frictions in cultural ecologies.

[50] Albers B, Metz A, Burke K, Bührmann L, Bartley L, Driessen P (2022). m.fl. The mechanisms of implementation support - findings from a systematic integrative review. Res Social Work Pract Marts.

[51] Metz A, Jensen T, Farley A, Boaz A, Bartley L, Villodas M. september. Building trusting relationships to support implementation: A proposed theoretical model. FrontHealth Serv. 23. 2022;2:894599. 10.3389/frhs.2022.894599 PMC10012819 36925800

[52] Powell BJ, Fernandez ME, Williams NJ, Aarons GA, Beidas RS, Lewis CC, m.fl. Enhancing the Impact of Implementation Strategies in Healthcare: A Research Agenda. Frontiers in Public Health [Internet]. 22. januar 2019 [henvist 1. juni 2020];7. Tilgængelig hos: https://www.frontiersin.org/article/10.3389/fpubh.2019.00003/full. 10.3389/fpubh.2019.00003 PMC6350272 30723713

[53] Gertner AK, Franklin J, Roth I, Cruden GH, Haley AD, Finley EP (2021). m.fl. A scoping review of the use of ethnographic approaches in implementation research and recommendations for reporting. Implement Res Pract Januar.

